# Evaluation of public’s knowledge, attitude and experiences towards the generic medications in building healthcare policy

**DOI:** 10.1371/journal.pone.0311627

**Published:** 2024-11-04

**Authors:** Md. Sayeed Akhtar, Khalid Orayj

**Affiliations:** Department of Clinical Pharmacy, College of Pharmacy, King Khalid University, Abha, Saudi Arabia; Lahore Medical and Dental College, PAKISTAN

## Abstract

The increasing cost of prescription drugs has high concern and associated with medication nonadherence and adverse health outcomes. The use of generic prescription drugs by the patients depends on if recommended by competent healthcare providers. Generic medicines, which are cheaper and bioequivalent to brand-name drugs, offer an opportunity to reduce healthcare expenditures of Saudi Arabia’s healthcare system as per the Vision 2030 plan. Therefore, the current study was conducted to assess the population awareness, attitude and perception towards the generic medications particularly those locally manufactured in Saudi Arabia. We managed a cross-sectional, questionnaire-based study and included indices like demographic sections, knowledge of generic medications, perception of generic medication use, and previous experience. Data was collected over 12 weeks from February 2022 to April 2022 using an online survey tool. A study of 462 participants found that the majority were male (54.97%), young (55.4%), with a graduate or post-graduate degree (73.5%) and employed (43.29%). The majority reported good health, with 75.5% reporting good health. The study found significant differences in knowledge about generic drugs, their country of origin, and their benefits and utility. Most participants had positive attitudes towards generic medications, with 45% believing they are as effective as those from other countries. The study also revealed a significant difference in participants’ experience and usage of medications particularly satisfaction to health outcome. Enhancing healthcare professionals’ perceptions of generics can positively influence patient/consumer opinions and trust in generic medicines. Patients’ high trust in prescribing physicians also influences these opinions, as their opinions can influence patient/consumer opinions.

## Introduction

The rising costs of pharmaceutical products indeed create a significant barrier to the accessibility and affordability of the healthcare services for the patients worldwide [1]. Saudi Arabia spends the most on healthcare in the Gulf Cooperation Council (GCC), and the highest share of tis gross domestic product (GDP) spent on healthcare [2]. Saudi Arabia’s healthcare system has recently inclined towards the government-private partnerships in a view to implement the Vision 2030 plan into action [2]. Generic medicines indeed play a crucial role in managing healthcare costs. They offer a more affordable alternative to branded drugs while maintaining the same effectiveness with the same ingredients. Hence, they provide an opportunity to curtail the escalating costs of medicinal products and reduce the burden on healthcare expenditures [3]. As per the United States Food and Drug Administration (USFDA), a generic drug is identical or bioequivalent to a brand name drug in dosage form, safety, strength, route of administration, quality, performance characteristics and intended use [4]. Generic medicines should be bioequivalent to the originator product, as well as similar in terms of strength, safety and quality. Perhaps, it may differ in colour, size, shape and excipients but possesses the same therapeutic effect as branded medicines at economic benefits [5]. Thus, generic drugs have been more and more popular to reduce pharmacoeconomic burden [6, 7]. Several studies have also shown that a sizable proportion of patients’ reporting negative views about generics, believing them to be less effective, of lower quality and unsuitable for the treatment of major illnesses, as compared to their branded equivalents [8–10]. These negative views could make a hurdle to the social adoption of generic medication. Multiple challenges hinder the broad use of generic medications worldwide, such as inadequate knowledge and negative perceptions as well as the lack of policies that enforce generic medication substitution [6, 8–12]. Consequently, the role of healthcare sector has become vital towards creating awareness regarding the generic medicines among the general population through various awareness programs regarding the use of generic medications [12–15]. In light of the current economic vision 2030 for the kingdom, there is an urgent need to bring the cost of the Saudi healthcare system to a sustainable level [16]. Hence, the substitution of branded drugs with their bioequivalent generics should offer a significant opportunity for cost saving without risking the quality of care. However, the use of generic medicines is restrained due to the limited knowledge and the negative perception of patients towards their quality, efficacy and safety [12, 17–19]. A Knowledge, Attitude and Practices (KAP) survey is a quantitative method having a pre-validated questionnaire to access quantitative and qualitative information. KAP studies are easy to conduct, measurable, and easily interpretable [20, 21]. There is a paucity of research regarding public knowledge and perception of generic medications in Abha, Asir province, Saudi Arabia. A better understanding of patients’ attitudes toward generics could assist in the development and targeting of educational campaigns to promote rational medication use. Keeping the facts of rising health burden over the decades, this study was undertaken to analyze the current public KAP about generic medications, particularly post-experiencing the locally manufactured generics when compared to the imported ones in Saudi Arabia. We also explored the correlation between knowledge, attitude and perception of generic drugs and sociodemographic variables. Concurrently, patient-provider communication about generics and patients’ comfort with generic substitution was also evaluated.

## Methodology

### Study design

A cross-sectional, questionnaire-based study was conducted to assess the public’s knowledge, perception and attitude about generic medications in Abha, Saudi Arabia. Convenience sampling was used by inviting people through various online platforms to participate in the study by filling out an online questionnaire.

#### Participants

Participants who can read English/Arabic and have the capability to complete an online survey were included in the study.

#### Outcome measures

An online questionnaire was developed and distributed to facilitate the data collection process. The outcome variables were composed of a demographic section (8 items) including included sex, age, marital status, level of education, health status. Another section included about the participants’ knowledge of generic medications (5 items), participants’ perception of the use of generic medications in general, and the locally manufactured versus (vs.) the imported generic medications in particular (10 items), and participants’ previous experience with generic medications (5 items) [12, 21]. Internal reliability of the knowledge and perception items was checked using Cronbach’s α statistics and was 7.83.

### Sample size

Epi info was used to calculate the required sample size, using the following equation. where Z is a standard normal variate (Z1−α/2 = 1.96 at 95% confidence interval (CI)), d is the absolute accuracy or precision (5% margin of error), and p is the expected proportion of the population with a specific outcome and was set at 0.5 (the advised value if the proportion in the population is not known). This yielded a required sample size of 385. We included 462 participants in our study to improve the validity of the data. Adults residing in Saudi Arabia at the time of the study were included. Exclusion criteria were being older than 80 years old with any mental, cognitive or severe psychiatric disorders that made them non-eligible to be part of the study

### Data collection

The data was collected over 12 weeks from February 2022 to April 2022, using an online survey tool for data collection and was secured within an encrypted database.

### Ethical approval

The ethical approval (Approval number: CM#2021–5909) was obtained from the Research Ethics Committee at King Khalid University (HAPO-06-B-001), Abha, Saudi Arabia. All study participants went through the statement of study consent before completing questionnaire.

### Statistical analysis

Descriptive statistics were applied to describe the participants’ demographics and their knowledge and perception of generic medications. Meanwhile, for inferential statistics, the Chi-square test was used to investigate differences in knowledge and perception of generics in different segments of participants. The internal reliability of the knowledge and perception items was checked using Cronbach’s α statistics. The data from responses to the Likert scale items were coded into numbers from 1 to 5 (totally disagree to agree) and transformed when necessary. The data were coded, checked for accuracy and analyzed using the IBM SPSS Statistics for Windows, version 25 (IBM Corp, Armonk, NY, USA).

## Results

A total of 462 questionnaires were completed by the participants. Most of the respondents were male (55.97%), of young age (<35 years ~55.4%), holding a graduate or post-graduate degree (73.5%), and were employed (43.29%). The respondents’ demographics were summarized in Table 1.

**Table 1:** Demographic characteristics of participants (N = 462).

Demographic characteristics	Number		Total
**Variables**	**Male (%)**	**Female (%)**	**Total**
**Gender**	254 (54.978)	208 (45.022)	462
**Age group (Years)**	**Male (%)**	**Female (%)**	**Total**
18–25	116 (76.32)	36 (23.68)	152
26–35	66 (63.46)	38 (36.54)	104
36–45	12 (16.22)	62 (83.78)	74
46–60	20 22.22)	70 (77.78)	90
61–70	40 (95.24)	2 (4.76)	42
**Marital Status**	**Male (%)**	**Female (%)**	**Total**
Married	100 (42.37)	136 (57.63)	236
Unmarried	154 (68.14)	72 (31.86)	226
**Educational level**	**Male (%)**	**Female (%)**	**Total**
High School	34 (48.57)	36 (51.43)	70
Diploma	32 (61.54)	20 (38.46)	52
Degree	148 (56.92)	112 (43.08)	260
Masters	24 (40.00)	36 (60.00)	60
Doctorate	16 (80.00)	4 (20.00)	20
**Current Job Status**	**Male (%)**	**Female (%)**	**Total**
Employed	112 (56.00)	88 (44.00)	200
Unemployed	104 (54.74)	86 (45.26)	190
Retired	38 (52.78)	34 (47.22)	72
**Health status**	**Male (%)**	**Female (%)**	**Total**
Healthy	206 (58.86)	144 (41.14)	350
Genetic disorder	2 (16.67)	10 (83.33)	12
One chronic disease	26 (48.15)	28 (51.85)	54
Multiple chronic disease	8 (33.33)	16 (66.67)	24
Not disclosed	12 (54.55)	10 (45.45)	22

N: Number of participants; %: Percentage

We observed a significant difference in 3 items regarding knowledge, namely “generic drugs have the same name and packaging of the original medicines (P<0.003), “generic drugs undergo the same quality and safety standards during manufacturing (P<0.001)” and “generic medicines differ from original medicines by containing some inactive elements such as colour and flavors (P<0.001)”. (Table 2A). As per the participants’ experience and usage of medications are concerned, most of the participants had positive experiences and usage of medications in all the domains indicating the acceptability of generic medications. A significant difference (P<0.001) was observed among the participants’ experience and usage of medications (Table 2B).

**Table 2:** Participants ’ knowledge/experience and usage of medications (N = 462).

2A. Participants ’ knowledge and usage of medications	YES (N)	%	NO (N)	%	P-Value
Generic drugs have same mechanism, of action and uses of the original medicines.	234	50.649	228	49.351	0.078
Generic drugs have the same dose and effects as of the original medicines.	212	45.887	250	54.113	0.077
Generic drugs have the same name and packaging of the original medicines.	200	43.290	262	56.710	0.003
Generic drugs undergo the same quality and safety standards during manufacturing.	316	68.398	146	31.602	0.001
Generic medicines differ from original medicines by containing some inactive elements such as color and flavors.	300	64.935	162	35.065	0.001
**2B. Participants’ experience and usage of medications**	**YES (N)**	**%**	**NO (N)**	**%**	**P-Value**
I have asked about the country of origin of the generic drug or searched for it on the package.	264	57.143	198	42.857	0.002
I have used generic medication in the past.	276	59.784	186	40.216	0.925
I am satisfied with the results, and I am thinking about using more generic medications in the future.	264	57.143	198	42.857	0.002

N: Number of participants; %: Percentage; P-Value less than 0.5 was considered as significant difference between the groups.

Table 3 depicts the participants’ beliefs about the value of the country of origin regarding the medications. The majority of the participants (approximately 45%) have responded positively for all the three items in the belief section, which were concerning about the use of generic medications, locally produced in Saudi Arabia and having comparable efficacy as other countries of origin. A significant difference (P<0.001) was observed among the participants regarding the beliefs about the importance of the country of origin for medications.

**Table 3:** Participants’ beliefs about the importance of the country of origin for medications (N = 462).

Participants’ beliefs about the importance of the country of origin for medications	Strongly Agree N (%)	Agree N (%)	Neutral N (%)	Disagree N (%)	Strongly Disagree N (%)	P-Value
Effectiveness of the drug may vary depending on the country of manufacture or the identity of the manufacturer (country of origin).	104 (22.5)	182 (39.4)	92 (19.9)	54 (11.7)	30 (6.5)	0.001
Locally produced generic medications are of high quality and can compete with western countries.	100 (21.6)	186 (40.3)	124 (26.8)	36 (7.8)	16 (3.5)	0.001
The quality and effectiveness of locally produced generic medications is superior to that of the manufactured in other countries.	66 (14.3)	134 (29)	168 (36.4)	74 (16)	20 (4.3)	0.001

N: Number of participants; %: Percentage; P-Value less than 0.5 was considered as significant difference between the groups.

Table 4 indicates the participants’ attitudes about the generic medications. The majority of the participants had a positive attitude for all the items which measured the benefits, utility and the acceptability of generic medications. A significant difference (P<0.001) was observed among the participants towards beliefs about the importance of the country of origin for medications.

**Table 4:** Participants’ attitude about the generic medications (N = 462).

Participants’ attitude about the generic medications	Strongly Agree N (%)	Agree N (%)	Neutral N (%)	Disagree N (%)	Strongly Disagree N (%)	P-Value
I think that generic medications can be as good as the brand medications that are considered as an alternative to them.	56 (12.1)	192 (41.6)	144 (31.2)	54 (11.7)	16 (3.5)	0.001
I think that generic medications can eliminate disease or control the symptoms as effectively as brand medications.	54 (11.7)	208 (45)	112 (24.2)	74 (16)	14 (3)	0.001
Generic medications have more side effects than the brand medications that are considered as alternative to them.	54 (11.7)	156 (33.8)	138 (29.9)	96 (20.8)	18 (3.9)	0.001
I always try to get generic medications instead of the original medications when it is available.	40 (8.7)	106 (22.9)	126 (27.3)	124 (26.8)	66 (14.3)	0.001
If the pharmacist dispensed to you a generic medication instead of the brand medications written in the prescription, you will accept the change that was made.	66 (14.3)	156 (33.8)	122 (26.4)	66 (14.4)	52 (11.3)	0.001
The use of generic medications helps in reducing the cost of the therapy without affecting the health of the patients.	72 (15.6)	172 (37.2)	124 (26.8)	62 (13.4)	32 (6.9)	0.001
I think it is better for doctors to prescribe more generic medications for their patients.	78 (16.9)	150 (32.5)	100 (21.6)	88 (19)	46 (10)	0.001

N: Number of participants; %: Percentage; P-Value less than 0.5 was considered as significant difference between the groups.

Table 5 shows the correlation between the age and education with participants’ knowledge and belief about medication. Data indicated that medication knowledge increased with age and higher education level is associated with more access to medication knowledge. We observed that there was a significant correlation between age and knowledge indices like ‘Generic drugs undergo the same quality and safety standards during manufacturing’ (P<0.001). In terms of participants’ beliefs about the importance of the country of origin of medications, we observed highly significant difference between the indices like ‘Effectiveness of the drug may vary depending on the country of manufacture or the identity of the manufacturer (P<0.001), ‘Locally produced generic medications are of high quality and can compete with western countries’ (P<0.001) and ‘the quality and effectiveness of locally produced generic medications is superior to that of the manufactured in other countries’ (P<0.1). However, the data indicated no significant correlation (P>0.01) between education level and participants’ knowledge and belief about medication.

**Table 5:** Correlation between age and education with participants ’ knowledge and belief about medications (N = 462).

Participants ’ knowledge and usage of medications	Age		Education	
	R	P-Value	R	P-Value
Generic drugs have same mechanism, of action and uses of the original medicines.	0.077	.099	0.015	0.750
Generic drugs have the same dose and effects as of the original medicines.	-0.023	.628	0.034	0.464
Generic drugs have the same name and packaging of the original medicines.	-0.089	.057	0.001	0.987
Generic drugs undergo the same quality and safety standards during manufacturing.	0.139**	.003	0.071	0.129
Generic medicines differ from original medicines by containing some inactive elements such as color and flavors.	-0.026	.575	-0.035	0.447
**Participants’ beliefs about the importance of the country of origin for medications**	**R**	**P-Value**	**R**	**P-Value**
Effectiveness of the drug may vary depending on the country of manufacture or the identity of the manufacturer (country of origin).	0.171**	0.000	0.038	0.421
Locally produced generic medications are of high quality and can compete with western countries.	0.183**	0.000	0.021	0.658
The quality and effectiveness of locally produced generic medications is superior to that of the manufactured in other countries.	-0.097*	0.038	-0.076	0.103

N: Number of participants; %: Percentage; R: Correlation Value; P-Value less than 0.5 was considered as significant difference between the groups.

In a similar fashion, Table 6 explains the correlation between age and education with participants’ attitude and experience about medications. We observed a significant correlation between age and attitude indices like ‘generic medications can eliminate disease or control the symptoms as effectively as brand medications’ (P<0.001), ‘Generic medications have more side effects than the brand medications that are considered as alternative to them’ (P<0.001), ‘acceptance of the generic medications in the place of branded medications if dispensed by the pharmacist’ (P<0.01), and ‘The use of generic medications helps in reducing the cost of the therapy without affecting the health of the patients’ (P<0.01). Considering the age and participants’ experience and usage of medications, significant correlations were observed for both the indices such as ‘usage of generic medication in the past’ (P<0.001), and ‘current satisfaction with the results of the generic medicines and decision to use more generic medications in the future’ (P<0.001).

**Table 6:** Correlation between age and education with participants ’ attitude and experience about medications (N = 462).

Participants’ attitude about the generic medications	Age		Education	
	R	P-Value	R	P-Value
I think that generic medications can be as good as the brand medications that are considered as an alternative to them.	0.050	0.282	-0.008	0.872
I think that generic medications can eliminate disease or control the symptoms as effectively as brand medications.	0.135**	0.004	0.103*	0.027
Generic medications have more side effects than the brand medications that are considered as alternative to them.	0.174**	0.000	-0.043	0.358
I always try to get generic medications instead of the original medications when it is available.	0.084	0.070	0.041	0.383
If the pharmacist dispensed to you a generic medication instead of the brand medications written in the prescription, you will accept the change that was made.	-0.115*	0.014	-0.219**	0.000
The use of generic medications helps in reducing the cost of the therapy without affecting the health of the patients.	0.104*	0.026	-0.020	0.664
I think it is better for doctors to prescribe more generic medications for their patients.	0.035	0.456	0.020	0.662
**Participants’ experience and usage of medications**	**R**	**P-Value**	**R**	**P-Value**
I have asked about the country of origin of the generic drug or searched for it on the package.	-0.086	0.065	-0.011	0.808
I have used generic medication in the past.	0.144**	0.002	-0.025	0.587
I am satisfied with the results, and I am thinking about using more generic medications in the future.	0.209**	0.000	-0.003	0.957

N: Number of participants; %: Percentage; P-Value less than 0.5 was considered as significant difference between the groups.

Considering the education level and participants’ attitude about the generic medications, data suggests a significant correlation in only two indices’ Generic medications have more side effects than the brand medications that are considered as alternative to them’ (P<0.01) and ‘acceptance of the generic medications in the place of branded medications if dispensed by the pharmacist’ (P<0.001). However, there was no significant (P<0.05) correlation observed between the education and participants’ experience and usage of medications.

## Discussion

Despite a good knowledge and a positive attitude among the patients/consumers documented by several researchers, a mistrust prevails amongst the users of generic medicines [8]. In facts, lack of information, awareness and education was a major issue in dispelling myths about generic medicines [10]. In our study, total of 462 participants completed the questionnaires to evaluate consumers’ knowledge, perception and attitude regarding generic medications.

Our study findings indicate that most participants were of young age (55.4%), male (55.97%) and holding higher education (73.5%),and spending healthy life (75.75%). Almost half of the participants in our study were employed. Nevertheless, the knowledge of participants regarding the generic medicines was different for various items of the knowledge domain. It is interesting to note that approximately 50% of participants are unaware of some crucial facts about generic medicines such as ‘Generic drugs have same mechanism of action and uses as of the original medicines’ and ‘Generic drugs have the same dose and effects as of the original medicines’. But it is promising that most of the participants were aware that the generic medicines have the same name and packaging of the original medicines (56.71%), they undergo the same quality and safety standards during manufacturing’ (68.39%), and they differ from original medicines by containing some inactive elements such as color and flavors (64.93%). This finding is supported by a similar study conducted by Almohammed et al. Study highlighted a general lack of knowledge regarding generic medicines [22]. Interestingly, 60% of the participants in their study exhibited a positive perception towards these medicines. These findings underscore the need for targeted interventions to enhance population awareness on matters related to generic drugs, particularly addressing concerns about their efficacy and safety. There is another study reporting that the community health practitioners are not confident of the curative efficacy and integrity profiles of generic medicine products [8, 23, 24]. Therefore, it is of utmost importance to take initiatives to improve understanding and trust in generic medicines to not only the public, but also to the healthcare practitioners by the Saudi Food and Drug Authority (SFDA). These initiatives could help in increasing the knowledge and the usefulness of the generic medicines among the public and the healthcare community leading to heightened use of generic medicines. It has been reported that Saudi Arabia imported MPP of around 2,078,407.217 USD in Dec 2022. Positively, data indicated that there could be a decrease in the import of medicinal and pharmaceutical products (MPP) than the previous years. [25]. In order to achieve the goals of Saudi Arabian Vision 2030, every citizen and the residents of Saudi Arabia should promote the use of locally manufactured products. It is observed from the results of our study that the participants are in favor of using the locally manufactured generic medicines, which evident from the responses of our participants stating that “Locally produced generic medications are of high quality and can compete with western countries (62%)”, and “The quality and effectiveness of locally produced generic medications is superior to that of the manufactured in other countries (43.3%)”. Unfortunately, more than half of the participants (62%) reported that the effectiveness of the drug may vary depending on the country of manufacture or the identity of the manufacturer. Thus, it is to be noted that this kind of notion among the public may hinder the achievement of the goals of the Saudi Arabian vision 2030. Interestingly, a study indicated that consumers have desire to discuss and understand more about the facts regarding the branded drugs and the generic drugs that counteracts the above discussed hindrances [26]. Therefore, creating awareness about the quality and effectiveness of locally produced generic medication among the public is of utmost importance as reported in previous study [27].

Certainly, it’s been a common perception that lower prices equate to inferior quality. However, its essential to debunk such myths and empower the consumers with accurate information through customer education, awareness programs, and credible endorsement to dispel the misconceptions regarding the generic counterparts [28]. In connection with attitude of the participants about the generic medications, our study reported poor attitude among Saudi population. Almost 53.7% and 56.7% of participants had positive attitudes regarding the items “Generic medications can be as good as the brand medications that are considered as an alternative to them” and “Generic medications can eliminate disease or control the symptoms as effectively as brand medications” respectively. However, concerning the side effects of the generic medicines, only 45.5% participants were having good clarity that there is no change in side effects between the generic and the branded medicines. Moreover, a small percentage of participants (31.6%) were reporting that they tried to get generic medications in place of the branded medications if available, which indicates the inclination of Saudi population towards the branded drugs. Further, if the pharmacists happened to dispense the generic drugs as a replacement for branded drugs, only 48.1% of participants had the will to accepts this change. The rest of the participants are tough enough to resist the interchange of brand medicines with their generic counterparts, which poses great challenge on pharmacists in convincing them. This may be due to either lack of knowledge or awareness and certainly their attitude, as evidenced in our study and other previous studies [29–31]. In line with above facts, around 53.1% accept that use of generic medications helps in reducing the cost of the therapy without affecting the health of the patients. The study participants (49.4%) think that it is better for doctors to prescribe more generic medications for their patients. Study also indicated inclination towards improvement in confidence regarding use of generic medicines as reported earlier in other studies [32]. In this context, active participation of physician in promoting the use of generic drugs is a definite way-out in acceptance of its usage. However, clinicians themselves are deprived in attitude and perception, thus general population concurrent to health care professionals need to be aware about therapeutic benefits of generic medicines comparing the branded ones [24, 33, 34].

We also observed the participants’ experience and usage of medications. The study indicated that around 60% of participants used generic medicine in the past. A similar percentage (57.143%) of participants either asked about the country of origin of the generic drug or searched for it on the package concurrent to their satisfactions [26, 35]. This further supports our above findings to take initiatives by SFDA.

Once evaluating the correlation between age and level of education with participants’ knowledge, attitude, and experience about medication, we found that both have major impact on usage of generic medications. The level of education and age maturity have great impact as reported in earlier studies [36]. Similar to previous studies, we also observed the influence of these age and higher educational levels were consistently more likely to have positive attitudes towards generics switch [12]. Thus, this is well evident that educational awareness programs should be implemented to improve the usage of generic medications.

## Conclusion

Enhancing the general public’s preconceived notions about generic drugs has the power to favorably alter their beliefs and level of confidence in use of generic drugs. Additionally, since patients show a high degree of trust in prescribing physicians and pharmacists, the opinions held by healthcare providers can impact patients’ insights. We recommend introducing mechanisms to acknowledge patients with enough information about the safety and effectiveness of generics through the general population awareness initiatives in the favour of generic medicines.
